# Nitric Oxide in Asthma Physiopathology

**DOI:** 10.5402/2011/832560

**Published:** 2011-04-19

**Authors:** Carla M. Prado, Mílton A. Martins, Iolanda F. L. C. Tibério

**Affiliations:** ^1^Department of Medicine, School of Medicine, University of São Paulo, 04023-900 São Paulo, SP, Brazil; ^2^Departmento de Ciências Biológicas, Universidade Federal de São Paulo, 04301-012, Diadema, SP, Brazil

## Abstract

Asthma is a chronic inflammatory airway disease characterized by allergen-induced airway hyperresponsiveness, airway inflammation, and remodeling. Nitric oxide (NO) derived from constitutive and inducible enzymes affects many aspects of asthma physiopathology. Animal *in vivo* studies have indicated that inhibition of iNOS may play a central role in the modulation of these features, particularly extracellular matrix remodeling. Additionally, increases in iNOS-derived NO, observed in asthmatic patients, may lead to an increase in peroxynitrite and an imbalance of oxidant and antioxidant pathways. In addition, endogenous nitric oxide produced by constitutive enzymes may protect against the remodeling of the lung. Therefore, nitric oxide donors and/or iNOS inhibitors may have therapeutic potential in asthma treatment and can also be used with corticosteroids to counteract airway remodeling. This paper focuses on the pathophysiological role of nitric oxide, mainly derived from inducible isoforms, in the various pathologic mechanisms of allergic asthma and the importance of nitric oxide and/or arginase inhibitors in asthma treatment.

## 1. General Aspects Regarding Nitric Oxide

Nitric oxide (NO) has long been considered an atmospheric pollutant released by cigarette smoke and vehicles [1] that contributes to ozone layer destruction [2]. In 1980, Furchgott and Zawadzki [3] observed that the presence of intact endothelial cells is essential to arterial relaxation induced by acetylcholine, because a relaxant factor that was endothelium-dependent was released. Because the nitric oxide half life is extremely short, it was not until seven years later when Palmer et al. [4] and Ignarro et al. [5] showed that this relaxant factor, which was found in veins and arteries, was a form of nitric oxide. Therefore, nitric oxide began to be recognized as an essential molecule to the function of various organs in the human body. 

Nitric oxide can be found in abundance in the central and peripheral nervous system [6, 7], acting as a classic neurotransmitter in the regulation of gastrointestinal motility, regional control of blood flux, and neuroendocrine function. In the brain, it affects memory formation and the response intensity to pain stimuli [8]. In addition, Kobzik et al. [9] demonstrated that nitric oxide is present in the skeletal musculature regulating its metabolism and muscle contractility. At high concentrations, it can have cytotoxic and cytostatic effects acting in the host defense against tumors and pathogens [10, 11].

NO has an unpaired electron, which makes it a free radical that can react with other molecules, such as oxygen, superoxide radicals, or transition metals [12]. Nitric oxide production begins when a group of L-arginine amino acid is broken down by oxidative enzymatic activity [13–16], generating nitric oxide and L-citrulline but not D-arginine [2, 10]. 

**Figure 1:**
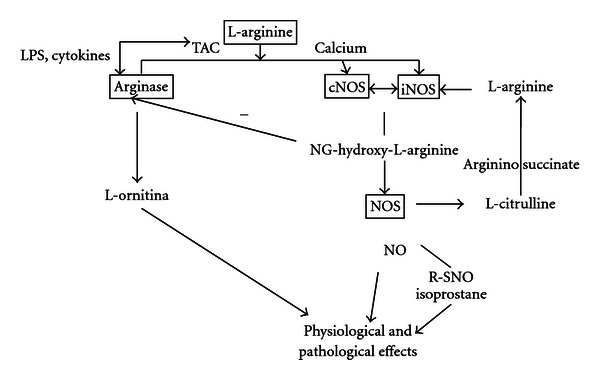
L-arginine, the common substrate to both the nitric oxide synthase and the arginase pathway. L-arginine is catalyzed by both arginase and nitric oxide. Arginase produces L-ornithine and urea that can act in various organs. In contrast, nitric oxide can be produced by both constitutive (cNOS) and inducible (iNOS) nitric oxide synthases and have pathophysiological roles important in health and diseases via the direct or indirect effects on oxidative stress production. Thus, arginase regulates the production of NO, and NO regulates the activity of arginase by substrate competition.

The L-arginine is transported inside the cells via a cationic amino acid transporter (CAT) and can be metabolized by two distinct groups of enzymes: nitric oxide synthase and arginase (Figure 1). Classically, arginase is an enzyme that acts in the urea cycle in the liver. However, this enzyme has been discovered in other cells that do not express the complete urea cycle, including the lung [17, 18]. 

The nitric oxide is generated during the conversion of the amino acid L-arginine to L-citrulline by nitric oxide synthase (NOS) using the NG-hydroxyl-L-arginine as an intermediate that in turn inhibits the arginase activity [19]. The L-citrulline can be converted from the argininosuccinate into L-arginine, and arginase is able to metabolize L-arginine to L-ornithine (Figure 1) [11]. There are three nitric oxide enzyme isoforms, two constitutive and one inducible: the neuronal or type I (nNOS) [7, 20], the inducible isoform or type II (iNOS) [21–23], and the endothelial isoform or type 3 (eNOS) [24]. All the isoforms are flavoproteins that have tetrahydrobiopterin, heme, and an area that is homologous to cytochrome P450 reductase. They act as dioxygenases using an oxygen molecule and nicotinamide adenine dinucleotide phosphate-oxidase (NADPH) to transform the L-arginine into L-citrulline and release the nitric oxide. The tetrahydrobiopterin (BH4) is the main cofactor for all nitric oxide synthases isoforms, and in its absence, these enzymes can produce superoxide instead of nitric oxide [25, 26].

Although the sequence of amino acids of iNOS from mice macrophages was similar to the sequence found in rat cerebellum cNOS (51% of identity), these represent distinct enzymes [21]. 

The nNOS and eNOS, which are constitutive, are normally present in physiological situations. The constitutive nitric oxide synthase isoforms are activated by the increase in intracellular calcium. However, some authors have found increases of these isoforms in inflammatory situations such as asthma [27, 28]. In addition, these enzymes are calcium-dependent and can produce low quantities of nitric oxide. The eNOS was first discovered in endothelial cells [24], and the nitric oxide formed by this isoform is responsible for maintaining low vascular tone and preventing the vascular wall adherence of leucocytes and platelets [16]. 

The nNOS isoform is present mainly in neurons [29] and nerves; however, it has also been found in airway smooth muscle cells [30] and in epithelial cells [31]. The nitric oxide derived from this isoform acts as a neuromodulator and/or neuromediator in the central and periphery nerves and in the termination of nerves in the nonadrenergic noncholinergic nervous systems, particularly as an iNANC mediator [32–35], causing bronchodilation in smooth muscle airways cells. Ward et al. [36] studied human lung-tissue and observed that iNANC relaxation depends on nitric oxide release being higher in the trachea and relatively lower in the distal airways.

In contrast, the lipopolysaccharides (LPSs) and other cytokines increase the L-arginine transport, inducing NO production by both inducible NOS (iNOS) and arginase activity. In addition, nitric oxide can react with thiol groups, releasing S-nitrosothiols (R-SNO) that induces a variety of physiologic effects mediated by oxidative/nitrosative stress.

However, the inducible nitric oxide is present mainly in pathophysiological situations, and its stimulation produces high quantities of nitric oxide. The presence of this isoform was demonstrated initially in macrophages; however, it is well known now that iNOS can act in a variety of tissues and organs including the vascular endothelium, inducing multiple functions in the response to inflammation [37–39]. iNOS is inducible by LPS and other cytokines, such as TNF-*α*, IL-1 and IFN-*γ*, stimulating the formation of nanomolar quantities of nitric oxide [10, 40]. 

Many studies have evaluated the presence of iNOS in different organs, and now it is well established that this isoform can be present in different types of cells including epithelial cells [41], macrophages [42, 43], neutrophils [42, 44], eosinophils [45, 46], and mononucleated cells [46].

Among the various functions of nitric oxide derived from iNOS, it is important to note the proinflammatory role of helping in the inflammatory cell recruitment to a local injury site. Another important role that has been intensively discussed is that nitric oxide produced by inflammatory cells can contribute to the amplification of the inflammatory response [12, 47, 48].

The NO production can be inhibited by the blockage of nitric oxide synthase enzyme [10] or by the administration of an alternative substrate that inhibits the production of NO by these enzymes. Recently, many NO inhibitors have become commercially available that block the action of these enzymes. Some examples of these inhibitors are L-NG-monomethyl-arginine citrate (L-NMMA), L-NG-nitroarginine methyl ester (L-NAME), NG-nitro-L-arginine (L-NOARG), NG-iminoethyl-L-ornithine (L-NIO), L-NG-nitroarginine-p-nitroanilide (L-NAPNA), L-canavanin, hydroxycobalamin and aminoguanidine [49].

Aminoguanidine was considered to be a specific inhibitor of iNOS [50]; however, recently, its specificity to block iNOS has been debated [28]. Some years ago, Garvey et al. [51] demonstrated that 1400 W presented a high selectivity to iNOS inhibition both *in vitro* as *in viv*o, presenting toxic effects only in high doses (25 mg/Kg). Therefore, the 1400 W has been used as a pharmacological tool to study the role of nitric oxide produced by iNOS in various pathophysiological processes, including bronchial asthma.

As previously demonstrated in Figure 1, the substrate L-arginine is a common substrate for nitric oxide synthase and arginase. The blockage of one of these pathways can affect the activation of the other by substrate competition [11, 52]. Some authors suggested that the blockage of nitric oxide by L-NAME could be considered a pharmacological strategy to study the indirect role of arginases in several diseases [18, 52].

## 2. NO in Allergic Asthma Physiopathology

During the last few decades, the role of nitric oxide has been studied in the physiopathology of different inflammatory diseases [28, 51, 53–62]. 

Barnes [63] first suggested that NO produced by nNOS has beneficial effects in asthma, causing bronchodilation by relaxing airway smooth muscle via NANC nerves activation. However, nitric oxide produced by eNOS can induce vasodilation in the arterioles, with plasma extravasation and edema. High quantities of nitric oxide produced by iNOS can result in vasodilation, plasma extravasation, increase in mucus secretion and indirect activation by Th2 cells (mainly due to eosinophilic recruitment) to contribute to asthma physiopathology. 

However, the role of nitric oxide in asthma is not to as simple as has been initially described because various *in vitro* and *in vivo* studies with animal experimental models and humans showed different results. The nitric oxide effects seem to be dependent on the animal mode and type of NO inhibitor as well as the parameters evaluated.

**Figure 2:**
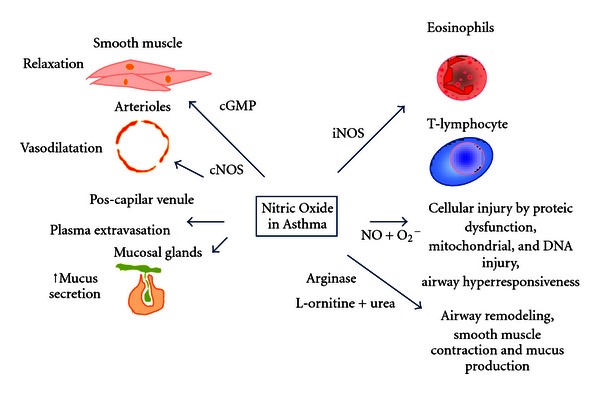
The dual effects of nitric oxide in asthma pathology. In asthma, nitric oxide can have both beneficial and adverse effects. The production of NO by constitutive isoforms can relax the smooth muscle of airways and vessels via cyclic GMP regulation inducing bronchodilation and vasodilation. However, by acting in the postcapillary venule, it can induce plasma extravasation. Nitric oxide can also regulate the mucosal glands, increasing the mucus secretion. High amounts of nitric oxide produced by iNOS in pathological situations induce the inflammatory cell chemotaxis, particularly recruiting eosinophils and T-lymphocytes to the lung. The reaction of nitric oxide with anion superoxide increases the oxidative stress pathway and can induce cellular injury by protein dysfunction or DNA injury and airway hyperresponsiveness. By substrate competition, nitric oxide can control the arginase pathway and induces airway remodeling, smooth muscle contraction and mucus production.

Some authors suggested that NOS activation can exert both beneficial and detrimental effects [18], such as altering relevant aspects of asthma pathology and participating in inflammatory cell recruitment [46–48, 55], airway responsiveness [52], and airway remodeling [11, 12, 64], as illustrated in Figure 2.

Nitric oxide was detected in the exhaled air in normal humans, but not in animals [65]. Various researchers demonstrated that the exhaled nitric oxide concentrations were increased in asthmatic patients [66–69] and in experimental models of chronic pulmonary inflammation [48, 70, 71]. Some authors suggested that the increase in exhaled NO can be due to increases in iNOS expression [22, 63, 66, 72]. It has been shown that L-NAME or aminoguanidine inhalation was able to reduce the amount of NO exhaled in healthy and asthmatic patients [73, 74]. Yates et al. [75] and Kharitonov et al. [72] noted that treatment with corticosteroids decreased the exhaled NO in asthmatics, but not in normal individuals. In addition, the exhaled NO was unaltered by the use of *β*_2_ agonists [76]. Recently, Dweik et al. [77] have shown that fractional exhaled nitric oxide can be clinical used for the identification of the most reactive asthma phenotype. In this regard, these authors showed that among patients with severe asthma, FeNO can identify the most reactive and the worst asthma phenotype.

 In contrast, Lim et al. [78] found no correlation between exhaled NO and the number of eosinophils found in airways, suggesting that NO does not directly modulate the eosinophilic inflammatory response in asthma. Thus, NO measurement should not be the only marker of disease control for asthmatic patients.

The exhaled nitric oxide has been proposed as an indirect marker of inflammation to be used in clinical practice. However, the efficacy of these measurements is a matter of controversy. Some authors showed that exhaled nitric oxide was a good marker of inflammation [79], which could be used to control the treatment of asthma and that it could be correlated with bronchial hyperreactivity in asthmatic children [80]. However, in a recently published study, Petsky et al. [81] showed that the tailoring of asthma treatment based on sputum eosinophils, but not in exhaled nitric oxide, was effective in decreasing asthma exacerbations. Despite these controversies, there is a consensus that exhaled nitric oxide is elevated in asthmatic patients, and repeated noninvasive and standardized monitoring contributes to the efficacy of the treatment of asthma because it is reduced in a dose-dependent manner by the use of inhaled corticosteroids [82].

## 3. Vascular Permeability

Nitric oxide plays an important role in controlling vascular tone [10]. There is evidence that vasodilation mediated by nitric oxide may promote edema formation via neural stimulation (see Figure 2). This mechanism is sensitive to treatment with tetrodotoxin, a drug that blocks calcium channels that are responsible for voltage-dependent propagation of neuronal action potential.

Nitric oxide modulates increased vascular permeability in the airways [83]. Ialenti et al. [84] showed that the inhibition of nitric oxide synthase by L-NAME, and L-NMMA inhibits the plasma leakage induced by carrageenan in rat skin and edema induced by carrageenan and dextran in the animal paw.

Erjefalt et al. [85] observed that NOS inhibitors decreased edema formation in the airway epithelium of guinea pigs. The NO-dependent changes in permeability may also be due to the release of other mediators, such as histamine, PAF and SP in the airways.

In an acute model, Mehta et al. [86] showed that chronic inhibition of nitric oxide synthase (72 hours) with L-NAME reduced plasma proteins. However, these results were not modified by aminoguanidine treatment, suggesting that vascular effects could be attributed, in particular, to constitutive isoforms.

Kuo et al. [87] suggested a link between substance P (SP) and NO production in airway microvascular permeability. Ziche et al. [88] demonstrated that SP can induce NO production by endothelial cells of postcapillary venules. Nguyen et al. [89] found that L-NAME blocks the increased vascular permeability induced by substance P, which was restored by L-arginine, the precursor of NO.

Our previous results [47, 48] showed that only chronic NO inhibition by L-NAME treatment attenuates the peribronchial edema formation around airways in an animal model of chronic allergic airway inflammation. In addition, when we evaluated the effects of specific iNOS inhibition in the same model, we did not observe any effects on plasma extravasation around airways, and in fact, this inhibition reduced the inflammation in the lung. Corroborating our findings that only cNOS played a role in the airway edema observed in asthma pathology, Bernareggi et al. [90] suggested that NO suppresses plasma leakage only in physiological situations and not when iNOS is highly expressed.

## 4. Airway Hyperresponsiveness

Donors of nitric oxide (nitrovasodilator) are smooth muscle relaxants, including airway smooth muscle *in vitro *[91, 92]. NO administration relaxes the smooth muscles of the trachea and airways and reduces methacholine-induced bronchoconstriction in guinea pigs and rabbits [93, 94]. Stimulation of nerve endings induces nNOS activation, and the NO release increases cyclic guanosine monophosphate (cGMP) levels in vascular smooth muscle inducing relaxation [10, 95].

The role of nitric oxide in the modulation of bronchoconstriction has been studied in animal models of both acute and chronic inflammatory responses. In acute models, Persson et al. [96] first showed that pretreatment with L-NAME increased inspiratory pressure, and exogenous nitric oxide administration was able to reverse this effect. Nijkamp et al. [97] showed that bronchoconstriction induced by intravenous administration of histamine in naïve guinea pigs was dose-dependently potentiated by L-NAME treatment. In addition, epithelium removal increased responsiveness to histamine. Other studies also suggested that the airway epithelium secretes nitric oxide [98].

Corroborating this hypothesis, De Sanctis et al. [27] observed that nNOS knockout mice led to a reduction in exhaled NO in both the basal and maximal response to methacholine responsiveness. De Boer et al. [99], studying isolated trachea of guinea pigs sensitized with ovalbumin, showed that endogenous nitric oxide deficiency induced by treatment with L-NAME increased bronchial responsiveness to methacholine after the immediate asthmatic reaction, which was reversed by L-arginine administration.

Although many studies emphasize the role of NO as a bronchodilator of the proximal airways, few studies have evaluated the role of NO as a modulator of distal airway tone. Dupuy et al. [93] found that inhaled NO can diffuse through the barrier of the bronchial epithelium and cause bronchodilation. In this study, the NO effects in the distal airways, which was measured by the complacency effect, was found only with high doses of inhaled nitric oxide, suggesting a larger role for this mediator in the proximal airways.

The importance of the distal lung in the pathophysiology of asthma has been evidenced recently in both in humans and in animal models [100, 101]. It has been suggested that small airway alterations may significantly contribute to the functional impairment in asthma, especially in the most severely affected patients [102, 103]. Our group showed that nitric oxide can act as a constrictor in areas other than the proximal airways. Both L-NAME and 1400 W reduced the lung-tissue mechanics in a model of inflammation induced by chronic challenges with ovalbumin [104, 105], suggesting that iNOS, which was probably produced by inflammatory cells that presented in the lung-tissue, regulated the constriction of the lung-tissue.

In contrast, some studies failed to show an important role of NO as a bronchodilator. Taylor et al. [106] studied patients with moderate persistent asthma and found that nebulization of L-NAME did not alter antigen-induced bronchoconstriction in patients with immediate and delayed responses. In this study, exhaled NO increased during the late response, and the L-NAME reduced exhaled NO levels by approximately 75%.

Another mechanism involved in the NO modulation of pulmonary mechanical alterations in asthma may be the interaction with the cholinergic neurotransmitters. In this regard, Belvisi et al. [34, 107] studied the effect of L-NAME on the NANC constriction response system by means of electrical stimulation in isolated tracheas of guinea pigs and humans. The results showed that L-NAME significantly increased cholinergic neurotransmission in a dose-dependent manner, but it was not effective in modulating constrictor responses of the NANC, suggesting that this effect was specific in cholinergic nerves.

Corroborating the interaction between nitric oxide and neurokinins, our group showed that capsaicin-sensitive sensory nerve fibers and neurokinins modulate nonneuronal nNOS expression by inflammatory and respiratory epithelial cells [108]. This increase in nNOS expression was also maintained for 14 days after capsaicin treatment and correlated with pulmonary mechanical reduction observed after capsaicin treatment when the lung content of neurokinin was already reduced [109]. 

Moreover, this study demonstrated that the acute inhibition of nitric oxide production with L-NAME treatment in sensitized mice restored the bronchodilator effects induced by neuropeptide depletion in both the proximal and distal airways [46].

Ricciardolo et al. [110] observed that the administration of L-NAME significantly increased the respiratory system resistance in response to the administration of bradykinin, but not to capsaicin in nonsensitized guinea pigs.

## 5. Leukocytes and Their Mediators

The modulation of the inflammatory process by NO production has been a matter of controversy. Nitric oxide may contribute to the recruitment of neutrophils, monocytes and eosinophils [23]. It is produced by some cells of the body's defense as neutrophils and macrophages [40]. 

Although the evidence from many studies has suggested that the inducible isoform is the major isoform involved in the inflammatory responses [42–45], recent studies suggest that the neuronal isoform also plays an important role in inflammation [111, 112]. It is known that there is eNOS and/or nNOS immunoreactivity in the various cells involved in inflammation, such as the lymphomononuclear cells [46], neutrophils [113, 114], eosinophils [46, 112, 115], and mast cells [116].

Nitric oxide may contribute to the extravasation of eosinophils from the circulation into the lung-tissue. Nitric oxide inhibition by L-NAME treatment does not influence eosinophilopoiesis [54].

Cardell et al. [117] showed that the inhibition of nitric oxide synthase reduces neutrophil recruitment and nasal secretion induced by LTB4 in the nasal mucosa of dogs. Trifilieff et al. [118] showed that iNOS promotes airway inflammation by increasing the expression of cytokines in an allergic asthma model in mice. 

Prado et al. [47] studied the effects of acute inhibition of nitric oxide production in a model of chronic airway inflammation in guinea pigs. The authors demonstrated that only acute L-NAME treatment reduces eosinophils and lymphocytes in airway walls of these animals. The majority of the lymphomononuclear cells were represented by CD4+ cells. The chronic treatment did not alter this response. The same authors also showed that iNOS inhibition, due to treatment with a high selective inhibitor, was able to reduce the eosinophilic and lymphocytic recruitment to airway walls in guinea pigs with chronic allergic pulmonary inflammation.

Regarding this issue, Kobayashi et al. [28] showed that acute treatments with L-NAME, but not 1400 W, attenuated eosinophilic recruitment. These differences could be related to compensatory increases in iNOS, with no effects on eosinophilopoiesis [119] or differences in apoptosis modulation [120]. Taylor et al. [120] suggested that NO could have different effects on inflammatory cell apoptosis (anti- and proapoptotic properties), depending on the concentration, flux, and source of NO.

McCluskie et al. [121] evaluated the effects of NO in an aerosolized LPS-driven animal model of airway inflammation. Their study involved the assessment of exhaled NO and the inflammatory response in mice treated with L-NAME (100 mg/kg) or 1400 W (30 or 100 mg/kg) 2 h before and 1 h after the challenge. Treatment with either drug resulted in a decrease in exhaled NO and airway neutrophilia. Iijima et al. [122] also evaluated the relationship between eosinophil recruitment and NO in sensitized mice using 1400 W (1.0 mg/kg) and L-NAME (10 mg/kg) administered 0.5 h before and 8, 20, 32, and 44 h after OVA challenge. The authors demonstrated that both L-NAME and 1400 W reduced the number of eosinophils in the bronchoalveolar lavage fluid.

In addition, eNOS-derived NO has been shown to inhibit airway inflammation by suppressing the activation of NF-*κ*B, thereby inhibiting both the expression of iNOS and the production of inflammatory cytokines [123–127].

Although there are discrepancies in the literature regarding the effects of nitric oxide in inflammation, there is a consensus that nitric oxide produced in high amounts has proinflammatory effects.

## 6. Airway Remodeling

Airway remodeling is characterized by structural changes in the airways and lung-tissue and involves hypertrophy and hyperplasia of the smooth muscle and goblet cells, extracellular matrix destruction and repair and neovascularization. Airway remodeling may be clinically important because it contributes to the irreversibility of lung function alterations observed in patients with asthma [128].

The role of nitric oxide in airway remodeling, particularly that associated with chronic inflammatory responses, has been recently emphasized [48, 129]. Gabazza et al. [130] showed a positive correlation between the concentrations of nitrite/nitrate in induced sputum from patients with asthma and bronchial remodeling, suggesting that NO is involved in the process of airway remodeling.

*In vitro* studies recently showed that DNA synthesis and proliferation of smooth muscle cells from human airways can be reduced by exogenous nitric oxide donors [30]. More recently, it was demonstrated that nitric oxide inhibits proliferation of smooth muscle cells of airways in G1 phase via cGMP-dependent mechanisms [131].

**Figure 3:**
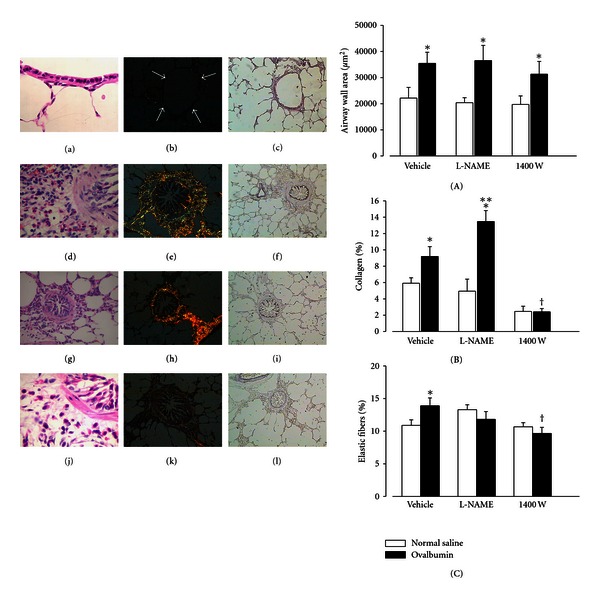
In the left panel, noncartilaginous guinea pig airways obtained from controls (a–c), ovalbumin-exposed (d–f), and ovalbumin-exposed animals treated with L-NAME (g–i) or 1400 W (j–l), a specific and highly selective iNOS inhibitor. We noted a weak yellow-greenish birefringence of the walls in the tissue section from control animals (b), coincident with the maintenance of the histoarchitecture of the extracellular matrix in H&E preparations (arrows) (a) and scant elastic fibers (c). In contrast, the airways of animals with airway inflammations induced by ovalbumin show an intense bronchoconstriction associated with peribronchial edema (d), an increase of birefringence in the airway wall (e) and in the elastic fibers content (f). L-NAME treatment decreased peribronchial edema (g), coincident with the increase of collagen content in the ECM (h), without interference in the elastic content (i). In contrast, 1400 W treatment attenuated the inflammatory cell infiltrate (j), collagen (k), and elastic (l) fiber deposition in airway walls without influencing peribronchial edema (original magnification in (b, c, e, f, h, i, k, l) ×200; (g) ×400; (a, d, j) ×1,000). In the right panel, the mean values of the total area (A), collagen (B), and elastic fibers (C) content in the airway walls of animals exposed to ovalbumin that received treatment with vehicle, L-NAME or 1400 W. *P < .05 compared with controls (open bars); **P < .05 compared with ovalbumin-exposed animals treated with vehicle and with 1400 W; and ^†^ *P* < .05 compared with ovalbumin-exposed animals treated with vehicle and L-NAME (closed bars). Reproduced with permission from Prado et al. [48].

As illustrated in Figure 3, we first demonstrated that nitric oxide derived from constitutive isoforms acts by protecting the extracellular matrix alterations in an experimental model of allergic airway inflammation, whereas iNOS-derived NO contributes to the increase of both collagen and elastic fibers [48]. Interestingly, the effects of nitric oxide counteracting the remodeling can also be observed in lung distal parenchyma [105]. 

Some authors have argued that arginase activation into myofibroblasts may act by increasing collagen production via production of L-ornithine [12, 18]. As previously described, arginase and nitric oxide synthase use a common substrate. Thus, chronic treatment with L-NAME is an indirect way to evaluate the role of arginase in the changes found in this model, particularly in airway remodeling. 

Hogaboam et al. [64] studied a model of nonfibrotic lung granuloma. They showed that L-NAME induces an increase in mRNA levels of the C-C chemokine receptors, CCR2 and CCR3, and reduces macrophage chemoattractants protein-1 and eotaxin in isolated lung fibroblast culture, increasing the collagen content. Previous reports suggested that arginase could interfere with myofibroblast differentiation, increasing collagen production. Meurs et al. [52] also suggested that allergen-induced deficiency of cNOS-derived NO enhanced arginase activity. Finally, NO can have some effects on metalloproteinase activation and expression to modifying collagen degradation [132].

Considering that airway remodeling is not totally controlled by current treatment, the nitric oxide pathway may be important for the therapeutic control of airway remodeling.

## 7. Activation of Oxidative Stress Pathway

Various enzymatic reactions and chemical processes can generate endogenous reactive oxygen species (ROS). Nitric oxide can interact with reactive oxygen species to form reactive nitrogen species (RNS). The oxygen and nitrogen reactive species as well as nitric oxide are crucial to many physiological functions in the body, mainly as defense mechanisms. However, when cells are exposed to oxidative stress, characterized by an imbalance between pro- and anti-oxidative molecules, they have a variety of deleterious effects. 

Some studies have also shown increased generation of superoxide radicals and ROS/RNS by alveolar macrophages (AMs), eosinophils, and polymorphonuclear leucocytes of asthmatic patients [133–135]. Both ROS and RNS produce many of the pathophysiological changes associated with asthma and may contribute to its pathogenesis. 

The manner by which the iNOS-derived NO production leads to the potentiation of lung-tissue mechanics is poorly understood, but it may be related to the promotion of peroxynitrite production. This oxidant agent is formed by the interaction of NO and superoxide, which leads to lipid peroxidation and isoprostane (8-epi-PGF2*α*) generation. PGF-2*α*, the main member of the family of isoprostanes, was produced via the peroxidation of arachidonic acid [136]. Isoprostanes contribute to smooth muscle contractions acting through tyrosine kinase, Rho and Rho kinase, leading to decreased activity of myosin light-chain phosphatase, which augments the level of phosphorylated myosin light-chain and contraction [136]. 

We previously showed that there was a significant decrease in 8-epi-PGF2*α* density in lung-tissue from guinea pigs that were repeatedly exposed to ovalbumin and treated with 1400 W [105]. These findings corroborate the notion that the iNOS-derived NO production increases the oxidative stress pathway, which may have detrimental effects on lung-tissue with functional repercussions.

The induction of chronic airway inflammation in guinea pigs resulted in an increase in oxidative stress. We evaluated the oxidative stress in this study by 8-iso-PGF2*α* staining [137]. The 8-iso-PGF2*α* is the most well-characterized isoprostane that may act through a novel receptor closely related to, but distinct from, the thromboxane A2/PGH2 receptor, with a high specificity for 8-iso-PGF2*α* [138]. Considering the physiological effects of isoprostanes, Quaggiotto and Garg [139] demonstrated that 8-iso-PGE2 produced physiological effects that were similar to 8-iso- PGF2*α*, but at a reduced potency. In addition, Wood et al. [138] showed that 8-iso-PGF2*α* was increased three-to-four-fold in patients with persistent asthma compared with the normal group. For these reasons, the evaluation of 8-iso-PGF2*α* represents a valuable indicator of the oxidative stress pathway [137, 140].

## 8. Future Directions and Clinical Applications of Nitric Oxide in Asthma

Although many studies using asthma animal models have shown beneficial effects associated with iNOS inhibition and/or maintenance of endogenous NO production by constitutive isoforms, in humans, this therapeutic approach has not been consistently established and must be further investigated.

Hansel et al. [141] showed a potent reduction in exhaled NO over a period of 72 h both in mild asthmatics and in healthy volunteers using the semiselective iNOS inhibitor L-NIL. Singh et al. [142] showed that treatment of asthmatic patients with a selective iNOS inhibitor (GW274150) reduced NO levels in patients, but had no effect on airway reactivity and inflammation in patients with asthma, suggesting that targeting NO may not be an effective intervention in asthma treatment. 

Considering that nitric oxide can have dual effects on asthma depending on the quantity, type of enzyme and location of release, it is important to consider the future drugs that may be donors of nitric oxide and/or inhibitors of nitric oxide, particularly the nitric oxide derived from iNOS. In this context, Turner et al. [143] showed in guinea pigs that TPI1020, a novel nitric oxide donating compound potentiates the bronchodilation activity of salbutamol. In addition, Jonasson et al. [144] showed that the association of nitric oxide and corticosteroids improved the protection against bronchoconstriction in an experimental murine model of asthma. The association of nitric oxide donors with corticosteroids and/or other current therapies used in asthma could be tested in clinical studies as airway remodeling therapies. 

Regarding the future clinical application of nitric oxide pathway, it is also important to consider the clinical use of arginase inhibitors. Arginases are enzymes that use a common substrate together nitric oxide, and various studies have shown that their activity is involved in airway remodeling. Maarsingh et al. [145] have shown that arginase inhibition attenuated chronic inflammation induced by antigen, particularly reducing the increase in smooth muscle mass, the airway bronchial reactivity, and the hydroxyproline content that is involved in lung fibrosis. The authors clearly suggested the emerging effectiveness of arginase inhibitors in the treatment of asthma.

## 9. Conclusions

Based on the results described above, we conclude that several studies of respected research groups around the world have suggested an important role of nitric oxide in the various mechanisms involved in the pathogenesis of asthma. They suggested that endogenous nitric oxide, produced mainly by constitutive isoforms, has protective effects on bronchoconstriction and on remodeling, whereas nitric oxide produced by iNOS in high amounts is involved in the constriction, inflammation and remodeling observed in asthma. Collectively, the results discussed in this paper suggest that iNOS inhibition or NO donors were promising therapeutic strategies for asthma treatment, particularly for counteracting the remodeling response. However, presently, commercially available drugs for asthma treatment do not affect this aspect of asthma pathophysiology.
